# Clinical application of an unshielded silicon diode detector for relative electron beam dosimetry

**DOI:** 10.1002/acm2.70682

**Published:** 2026-07-07

**Authors:** Teresa J. Anders, Veronika Flatten, Mohamad Alissa, David Towle, Olivier Evrard, Gerhard Wessing, Charbel Habib, Damian Czarnecki, Andreas A. Schönfeld

**Affiliations:** ^1^ Ärztepartnerschaft Radiologie Vechta Vechta Germany; ^2^ Universität Bremen Bremen Germany; ^3^ Sun Nuclear Corp Melbourne Florida US; ^4^ CDT‐West ‐ Centrum für Diagnostik und Therapie Cologne Germany; ^5^ Mirion Technologies Olen Belgium; ^6^ MyMichigan Health Midland Michigan USA; ^7^ Institut für Medizinische Physik und Strahlenschutz Technische Hochschule Mittelhessen Gießen Germany

**Keywords:** dosimetry, electron beams, silicon diode, unshielded diode

## Abstract

**Background:**

Relative electron beam dosimetry requires careful consideration of detector‐specific effects, including material dependent stopping‐power ratios, effective point of measurement, and volume averaging. Ionization chambers require percentage depth ionization (PDI) to percentage depth dose (PDD) conversion, while suitable silicon diode detectors may directly measure absorbed dose due to their more water‐equivalent stopping‐power behavior.

**Purpose:**

This study aims to evaluate the unshielded silicon diode detector SunSILICON 1048 (Sun Nuclear) for clinical electron beam dosimetry by assessing its suitability for measurement of PDDs, lateral beam profiles, and output factors.

**Methods:**

Comparative measurements between SunSILICON and different types of ionization chambers (SNC350p, SNC125c, SNC600c, all Sun Nuclear Corp.), as well as PTW's silicon diode detector microSilicon were taken on a Varian TrueBeam linear accelerator using a SunSCAN 3D water phantom for electron beam energies from 6 MeV to 22 MeV and field sizes from 6 cm × 6 to 25 cm × 25 cm. Ion chamber PDIs were converted to PDDs; silicon detector PDIs were evaluated directly. The agreement between repeated measurements conducted with different detectors was assessed using 1D gamma criteria.

**Results:**

SunSILICON PDDs agreed with SNC350p derived PDDs within ± 1% for all energies except in the 6 MeV buildup region. Gamma passing rates exceeded 94.5% except for 6 MeV, where deviations were confined to the buildup region. Lateral profiles showed gamma passing rates exceeding 96.7%, with differences dominated by ion chamber volume averaging. Comparisons with microSilicon exceeded 99% agreement for both PDDs and lateral profiles. Output factors matched SNC600c within 0.85% RMS.

**Conclusions:**

SunSILICON provides accurate, direct measurements of PDDs, lateral beam profiles, and output factors. Its water‐equivalent design minimizes perturbation and gradient related errors, confirming its suitability for clinical electron beam dosimetry.

## 1 INTRODUCTION

The challenge of relative dosimetry in clinical electron beams lies in the correct application and the full understanding of the chosen detector. The methodology is described in several dedicated national and international dosimetry protocols.^1^, ^2^, ^3^, ^4^, ^5^

For electron reference dosimetry using ionization chambers, it is necessary to know the $R_{50}$ depth, which is the depth at which the percentage depth dose (PDD) reaches 50% of its maximum, for each radiation quality to correctly position the detector.^6^, ^7^, ^8^

**FIGURE 1:**
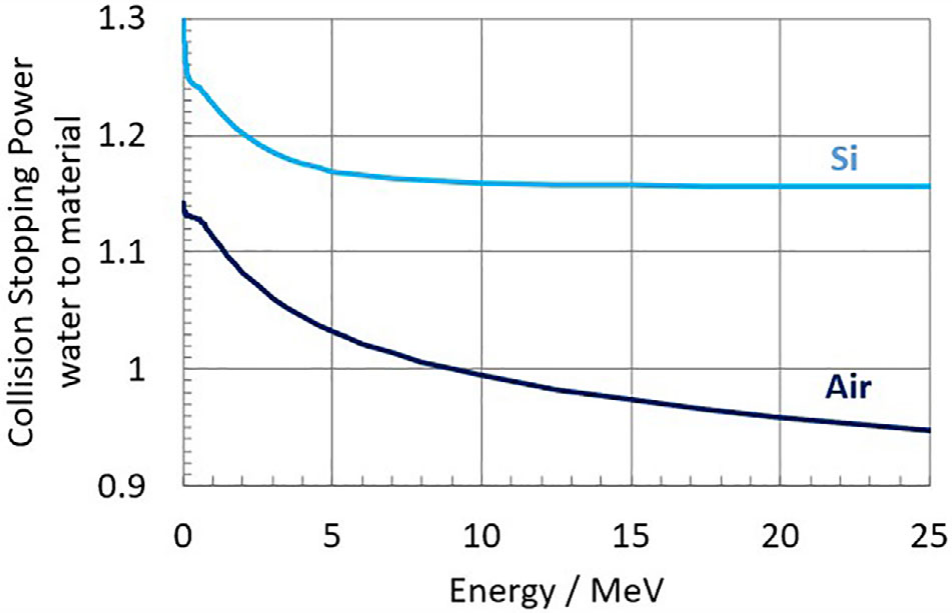
Unrestricted mass collision stopping‐power for mono‐energetic high‐energy electron radiation normalized to that of water in the energy range of 10 keV to 25 MeV (from^15^).

While ionization chambers are used for reference dosimetry, they bare inherent challenges in relative dosimetry. A depth scan conducted with an ionization chamber yields percentage depth ionization (PDI) values. The consequence is the necessity of converting PDI measurements to PDDs. Compared to photon dosimetry, where the mean photon energy, and thus the mean electron energy,^9^ is fairly constant with respect to depth in water,^10^, ^11^ electron beams are subject to continuous energy loss with depth. A rough estimate for clinical electron beam energies is about 2 MeV of energy transferred per cm of water.^12^, ^13^ Due to this energy gradient, the restricted mass collision stopping‐power ratios between water and the absorbing detector materials, denoted as ${\left(\bar{L}/\rho \right)}_{det}^{water}$, are much more relevant.^11^ Notably, ${\left(\bar{L}/\rho \right)}_{det}^{water}$ depends on the electron fluence spectrum at the point of measurement, which can be derived from Monte Carlo simulations.^11^, ^14^ The consequence is the necessity of converting PDI measurements to PDDs using

(1) $$\begin{array}{c} PDD\left(z\right)=PDI\left(z\right)\cdot \frac{{\left(\bar{L}/\rho \right)}_{air}^{water}\left(R_{50},z\right)\cdot P_{fl}\left(E_{d}\right)}{{\left(\bar{L}/\rho \right)}_{air}^{water}\left(R_{50},z_{max}\right)\cdot P_{fl}\left(E_{d_{max}}\right)} \end{array}$$

where ${\left(\bar{L}/\rho \right)}_{air}^{water}$ changes with energy and thus, with depth in water.^1^ Notably, electron fluence correction factors $\frac{P_{fl}\left(E_{d}\right)}{P_{fl}\left(E_{d_{max}}\right)}$ are unity for parallel plate ionization chambers.^1^ To illustrate the energy dependence of ${\left(\bar{L}/\rho \right)}_{air}^{water}$ in good approximation and guide the discussion of the observations made in this study, Figure 1 shows the simplified case of the unrestricted mass collision stopping‐power ratios in monoenergetic beams.^15^ A detailed analysis of ${\left(\bar{L}/\rho \right)}_{air}^{water}$ for clinical electron beams is presented by Ding et al..^11^ Since Equation 1 is not easily applicable in clinical practice, current dosimetry protocols reference a multi‐parameter conversion function published by Burns et al.^14^ to determine ${\left(\bar{L}/\rho \right)}_{air}^{water}\left(R_{50},\;z\right)$. The validity of the Burns equation under consideration of ICRU90^16^ has been verified in IAEA's TRS 398 Rev. 1.^6^

Secondly, ionization chambers are subject to perturbations which depend on the measurement depth caused by the density of the air‐filled sensitive volume and detector components (namely the chamber wall material). With increasing depth, the balance between electrons scattering into and out of the sensitive volume is disturbed, resulting in a systematic deviation of the measured PDD from the actual depth dose curve in water. According to dosimetry protocols this effect can be reduced by a shift of the effective point of measurement (EPOM).^5^, ^7^ However, several studies have shown that the EPOM shift is not only energy but also ionization chamber model dependent.^17^ As demonstrated by Zink et al.,^18^ wider collecting electrodes or guard rings, can mitigate but not eliminate these effects. Thus, depth‐dependent perturbation of the PDD cannot be avoided.

In contrast, the mass collision stopping‐power ratios of silicon to water are consistent across a broad range of clinically relevant electron energies (Figure 1) and adequate detector sensitivity can be achieved with very small sensitive volumes due to silicon's high density as well as the low ionization energy.^19^

This enables properly housed silicon‐based detectors to measure electron beam PDDs, lateral beam profiles and output factors directly, as is recommended by national and international dosimetry protocols.^1^, ^2^, ^3^, ^4^, ^6^, ^7^, ^8^ Notably, the applicability of silicon‐based detectors cannot be generalized. IAEA's TRS 398,^6^ AAPM's applicable TG reports^1^, ^2^, ^7^, ^8^ and IPEM^4^ emphasize the need to evaluate a detector model prior to its use, since the housing design can introduce significant fluence perturbations causing erroneous measurements. This is specifically true for shielded diode designs, where the high‐density shielding material significantly perturbates the electron fluence with increasing water depth.^4^

To date, no published data is available assessing the performance of the new unshielded 1048 SunSILICON detector (Sun Nuclear Corp., Melbourne, USA) for electron beam dosimetry, although the detector exhibits several promising characteristics. Specifically, its housing is engineered to be water‐equivalent, and the linear response behavior of the custom designed silicon diode has been confirmed with respect to both dose and dose rate, including variations in dose per pulse and pulse repetition frequency^20^. Thus, the aim of this study is to investigate the performance of the unshielded 1048 SunSILICON detector (Sun Nuclear Corp., Melbourne, USA) in clinical electron beam dosimetry.

## 2 MATERIALS AND METHODS

### 2.1 SunSILICON detector

The SunSILICON detector contains a p‐type, guarded silicon diode with an active volume of 0.053 mm^3^. The active volume has a radius of 0.75 mm and a depth of 0.03 mm. The diode operates without bias voltage. The detector housing is made of the water‐equivalent material HE Solid Water (Sun Nuclear Corp., Melbourne, USA) and epoxy. The detector was designed to minimize beam perturbation by surrounding the silicon diode disc only by water‐equivalent materials and moving the electronics further down the detector stem. The entrance window is perpendicular to the detector's long axis, and its nominal water‐equivalent build‐up depth is 1.25 mm. As is common for unshielded silicon diode detectors, SunSILICON is intended for use in clinical electron beams and small photon beams. The electron field size range specified by the manufacturer is 1 to 40 cm. Field sizes are expressed in terms of the width of the square field.

**FIGURE 2:**
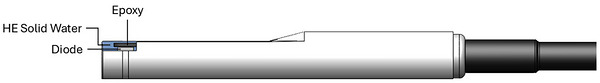
Drawing of the cross section of the unshielded 1048 SunSILICON detector. Not to scale, not all details shown.

A detector drawing of SunSILICON is shown in Figure 2. SunSILICON's general response characteristics were investigated in previous publications.^20^, ^21^, ^22^

### 2.2 Measurement setup

A Varian TrueBeam (Varian Medical Systems, Palo Alto, USA) clinical linear accelerator (linac) with nominal electron beam energies of 6, 9, 12, 16, 18, 20 and 22 MeV served as the irradiation device. All measurements were conducted with a SunSCAN 3D motorized water phantom (Sun Nuclear Corp., Melbourne, USA) using a source‐to‐surface‐distance (SSD) of 100 cm. Detectors were set up according to the manufacturer's setup recommendations using matching holders. Sun Nuclear's parallel plate ionization chamber 1045 SNC350p, thimble ionization chambers 1041 SNC125c and 1047 SNC600c, and PTW's silicon diode detector 60023 microSilicon (PTW, Freiburg, Germany)^23^ were used for comparative measurements.

### 2.3 Measurements

Electron beam PDIs, lateral beam profiles and field output factors were obtained for field sizes of 6, 10, 15, 20 and 25 cm at nominal beam energies from 6 to 22 MeV.

A SunSILICON, a microSilicon and a SNC350p detector were used to acquire a total of 19 PDIs per detector. PDIs measured with ion chambers were converted to PDDs following the Burns equation^14^, ^24^ using the SunDOSE software (Sun Nuclear Corp., Melbourne, USA). Following current electron dosimetry protocols,^1^, ^2^, ^3^, ^4^ no conversion was applied to PDIs measured with the investigated silicon diode detectors to evaluate their ability to directly measure PDDs.

The detectors were positioned with their respective reference points at the reference depth $d_{ref}$ to obtain lateral beam profiles and field output factors. The reference points of the cylindrical ionization chambers SNC125c and SNC600c is their central axis and that of SunSILICON is the center of its sensitive volume. The reference depth was determined with

$$\;d_{ref}=0.6\;R_{50}\;-\;0.1\;\mathrm{cm}$$

where $R_{50}$ was derived from PDIs measured with SNC350p using

$$\;R_{50}=1.029\;I_{50}-0.06\;\mathrm{cm}$$

For the plate parallel chamber SNC 350p, the front face of the air cavity is the effective point of measurement used for the PDI measurements in accordance with AAPM WGTG51 Report 385.^7^

Lateral beam profiles were measured with SunSILICON, microSilicon and SNC125c.

## 3 RESULTS

**FIGURE 3:**
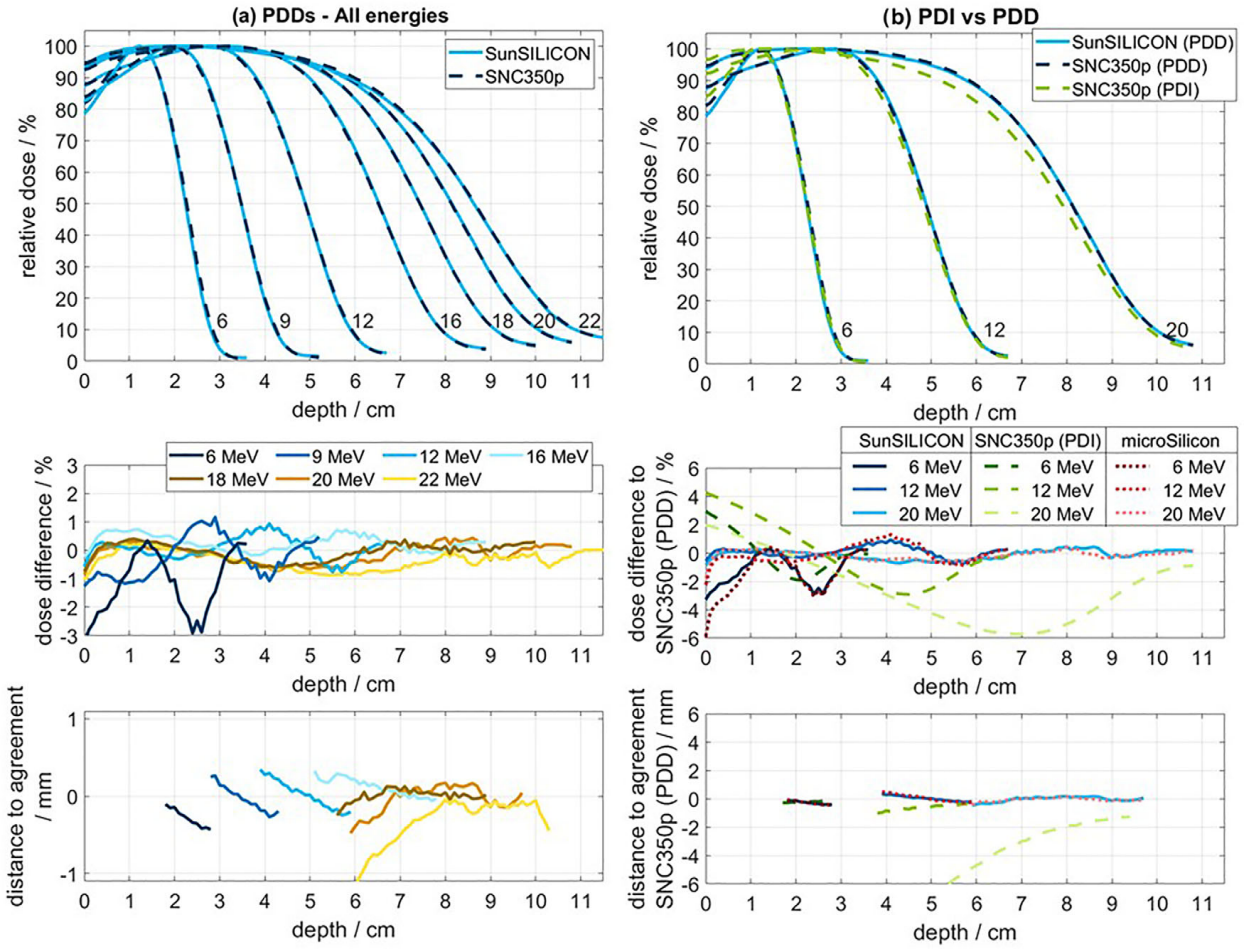
(a) Comparison of electron PDD measurements with SunSILICON and SNC350p at 10 cm field size. (b) Comparison of PDI and PDD measurements at 10 cm field size. Center row shows corresponding difference between measurements. Bottom row shows the distance to agreement. The distance to agreement is only shown from $R_{90}$ to $R_{P}$. (b) Middle row illustrates the differences between PDDs and PDIs measured with an SNC350p parallel plate chamber, which, in accordance with the evolution of the restricted mass stopping‐power ratios, increases with beam energy.^11^ Notably, the corrections needed with ionization chamber measurements are subject to uncertainty. However, the comparison to corrected ionization chamber measurements is recommended in dosimetry protocols and considered state of the art.^1^, ^2^, ^3^, ^4^, ^6^, ^7^, ^8^ ^.^

Representative PDD comparisons between SunSILICON and SNC350p for beam energies ranging from 6 to 22 MeV are shown in Figure 3a. The relative difference is less than about ± 1%, except in the build‐up region of the 6 MeV beam. The difference diminishes with increasing beam energy. The distance to agreement shown in the bottom row of Figure 3 is within ± 0.5 mm, except for the 22 MeV PDD, which is within ± 1 mm. The distance to agreement is restricted to the region between the distance where the dose falls off to 90% $R_{90}$ and the practical range $R_{P}$ because in a plateau, minor dose differences would result in major distance discrepancies. This effect can be observed for the higher energies, especially the 22 MeV curve, where the dose difference is always less than 1% but due to the small gradient the distance to agreement is significantly deviating. Applying a 1D gamma criterion of 1% / 1 mm and a low dose threshold of 10% yields passing rates above 94.5% for all measured PDDs, except for the 6 MeV beams. In all cases, deviations were confined to the build‐up region, which makes up a significant portion of the 6 MeV PDDs.

The comparison of a subset of PDDs with corresponding PDIs is illustrated in Figure 3b. Notably, silicon diode measurements and PDDs obtained from the SNC350p's PDI measurements agree within ± 1%, while the PDDs and PDIs differ by up to ± 6%. The difference between PDDs and PDIs significantly increases with beam energy.

**FIGURE 4:**
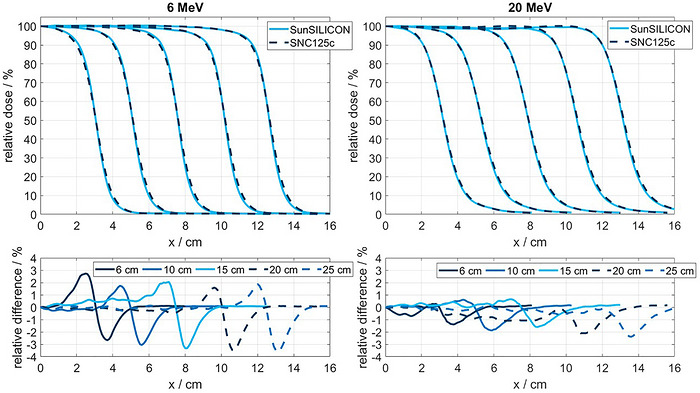
Comparison of lateral beam profiles measured with SunSILICON and the SNC125c ion chamber for 6 MeV electrons (left) and 20 MeV electrons (right). The top panels show half‐profiles for both detectors across five field sizes from 6 to 25 cm. The bottom panels show the difference between the two measurement sets, calculated by subtracting the SNC125c relative dose values from the corresponding SunSILICON values.

Figure 4 presents lateral beam profiles of 6 and 22 MeV beams as measured with SunSILICON and SNC125c. Applying the same gamma criterion to all measured lateral beam profiles yields passing rates above 96.7%, where the largest differences are observed at 6 MeV.

PDD and profile measurements repeated with PTW's microSilicon were intercompared using a stricter gamma criterion of 1% / 0.5 mm and no low dose threshold given that both detectors are of the same detector type. The passing rates were 100% with only three exceptions showing passing rates above 99%. A representative subset of dose difference and distance to agreement values is shown in Figure 3b.

**FIGURE 5:**
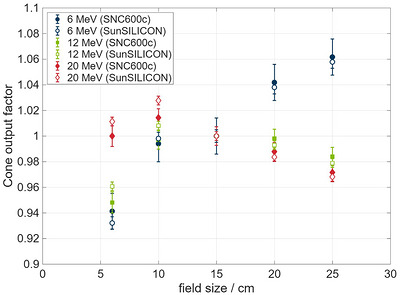
Exemplary cone output factors measured with SNC600c and SunSILICON at nominal beam energies of 6 MeV, 12 MeV and 20 MeV.Normalization was performed at the 15 cm field size as this is the center value and typical field sizes for reference dosimetry are 14 –20 cm square. The error bars indicate the setup and measurement uncertainty.

The comparison of cone output factors measured with SunSILICON and SNC600c (Figure 5) showed a root‐mean‐square‐deviation of 0.85% with no significant trend across all measured field sizes and energies.

## 4 DISCUSSION

The steepest gradients of the mean electron energy occur in PDD measurements, making PDD comparisons a meaningful tool to evaluate a detector. Evidently, measurements conducted with SunSILICON match the PDDs derived from PDI measurements with SNC350p (Figure 3a) indicating that fluence perturbations introduced by the unshielded detector housing are sufficiently small within the clinically relevant energy range. This aligns with the conclusion drawn from a previous fluence perturbation factor analysis in context of small photon field dosimetry.^21^ Since SunSILICON is linear with dose and dose rate^20^ and all measurements support by their agreement with references that the energy dependence is sufficiently small, the detector can be used to directly measure PDDs.

The further comparison to another unshielded silicon diode detector claimed to be suitable for electron dosimetry^23^ yielded near indistinguishable lateral beam profiles and PDDs in the fall‐off region beyond $D_{max}$ (Figure 3b). The correlation of SunSILICON and microSilicon measurements extends into the build‐up region, where silicon diode and ion chamber measurements deviate at low beam energies. In all cases, PDDs measured with SunSILICON were in better agreement with the PDDs derived from the SNC350p measurements. Akino et al.^23^ reported that the mean electron energy of a nominal 6 MeV beam drops below 5 MeV after only a few millimeters of water. It was thus concluded to have an impact on measurements in the build‐up region of a 6MeV electron beam because the stopping‐power ratio of silicon and water begins to be non‐linear below that energy (Figure 1). Other potential sources of uncertainty are the different inherent build‐up of the detectors, setup uncertainty, or uncertainty of the PDI and PDD conversion and underlying methodology of determining the applicable stopping‐power ratios.^11^

The distance to agreement plots in the bottom row of Figure 3 show that relevant electron beam parameters, such as $R_{50}$, $R_{20}$, $R_{80}$, $R_{p}$ and $D_{max}$, determined either directly with SunSILICON or indirectly with SNC350p are within ± 0.5 mm from each other, which is well within the tolerance of ± 1 mm set by AAPM's TG 142 for constancy checks of $R_{50}$ conducted with the same detector.^25^, ^26^

For lateral beam profile measurements, the notable differences observed in Figure 4 can primarily be attributed to the enhanced volume averaging effect of the SNC125 ionization chamber. Since the penumbra broadens with increasing electron energy, the magnitude of the volume averaging effect diminishes and a better agreement between the SNC125c and SunSILICON is observed at higher beam energies. When accounting for the difference caused by volume averaging via an estimation proposed by Kawrakow,^27^ the differences between the measurements are below 1% for all energies. A similar correlation between energy and detector agreement is observed for the output factor measurements (Figure 5), where the variation of the output factors decreases from more than ± 6% at 6 MeV to ± 3% at 20 MeV. The root‐mean‐square difference of the SNC600c and SunSILICON are within 0.85% and do not vary with energy.

In accordance with IAEA's TRS 398^6^ and AAPM WGTG 51: Report 385,^7^ the results of this study confirm that the unshielded SunSILICON detector is appropriate for clinical electron beam dosimetry.

## 5 CONCLUSION

This study confirms that the unshielded SunSILICON detector is suitable for clinical electron beam dosimetry. Its water‐equivalent housing and high spatial resolution allow direct measurement of lateral profiles, output factors, and PDDs. Using just a silicon diode simplifies setup and reduces complexity compared to mixing parallel plate and thimble chambers with PDI to PDD conversion.

## AUTHOR CONTRIBUTION

T. Anders validated measurements, analyzed data and drafted the manuscript. V. Flatten contributed to the design of the study analysis and substantially revised the manuscript. M. Alissa contributed to the detector design. D. Towle and C. Habib conducted the measurements on the Varian linac. O. Evrard designed and tested the diode. G. Wessing collected the measurements together with T. Anders. D. Czarnecki contributed to the study and detector design and substantially revised the manuscript. A.A. Schönfeld supervised the study design and analysis and substantially revised the manuscript. All authors revised the manuscript and approved the final version.

## CONFLICT OF INTEREST STATEMENT

Andreas A. Schönfeld, Veronika Flatten, Olivier Evrard, and David Towle are employees of Mirion. The remaining authors declare that the research was conducted in the absence of any commercial or financial relationships that could be construed as a potential conflict of interest.

## ETHICS STATEMENT

This study did not involve human participants or animals. All experiments were performed using phantoms and simulation data; therefore institutional review board approval and informed consent were not required.

## Data Availability

The data that support the findings of this study are available from the corresponding author upon reasonable request.
